# Erroneous computer-based interpretations of atrial fibrillation and atrial flutter in a Swedish primary health care setting

**DOI:** 10.1080/02813432.2019.1684429

**Published:** 2019-11-04

**Authors:** Thomas Lindow, Josefine Kron, Hans Thulesius, Erik Ljungström, Olle Pahlm

**Affiliations:** a Department of Clinical Physiology, Växjö Central Hospital, Växjö, Sweden;; b Department of Research and Development, Region Kronoberg, Växjö, Sweden;; c Department of Clinical Physiology, Division of Clinical Sciences, Lund University, Lund, Sweden;; d Department of Medicine and Optometry, Linnaeus University, Växjö, Sweden;; e Department of Cardiology, Section of Arrhytmias, Skåne University Hospital, Lund, Sweden

**Keywords:** ECG, atrial fibrillation, atrial flutter, computer-based interpretation, cardiovascular disease

## Abstract

**Objective:** To describe the incidence of incorrect computerized ECG interpretations of atrial fibrillation or atrial flutter in a Swedish primary care population, the rate of correction of computer misinterpretations, and the consequences of misdiagnosis.

**Design:** Retrospective expert re-analysis of ECGs with a computer-suggested diagnosis of atrial fibrillation or atrial flutter.

**Setting:** Primary health care in Region Kronoberg, Sweden.

**Subjects:** All adult patients who had an ECG recorded between January 2016 and June 2016 with a computer statement including the words ‘atrial fibrillation’ or ‘atrial flutter’.

**Main outcome measures:** Number of incorrect computer interpretations of atrial fibrillation or atrial flutter; rate of correction by the interpreting primary care physician; consequences of misdiagnosis of atrial fibrillation or atrial flutter.

**Results:** Among 988 ECGs with a computer diagnosis of atrial fibrillation or atrial flutter, 89 (9.0%) were incorrect, among which 36 were not corrected by the interpreting physician. In 12 cases, misdiagnosed atrial fibrillation/flutter led to inappropriate treatment with anticoagulant therapy. A larger proportion of atrial flutters, 27 out of 80 (34%), than atrial fibrillations, 62 out of 908 (7%), were incorrectly diagnosed by the computer.

**Conclusions:** Among ECGs with a computer-based diagnosis of atrial fibrillation or atrial flutter, the diagnosis was incorrect in almost 10%. In almost half of the cases, the misdiagnosis was not corrected by the overreading primary-care physician. Twelve patients received inappropriate anticoagulant treatment as a result of misdiagnosis.

**Key points**
- Data regarding the incidence of misdiagnosed atrial fibrillation or atrial flutter in primary care are lacking. In a Swedish primary care setting, computer-based ECG interpretations of atrial fibrillation or atrial flutter were incorrect in 89 of 988 (9.0%) consecutive cases.
- Incorrect computer diagnoses of atrial fibrillation or atrial flutter were not corrected by the primary-care physician in 47% of cases.
- In 12 of the cases with an incorrect computer rhythm diagnosis, misdiagnosed atrial fibrillation or flutter led to inappropriate treatment with anticoagulant therapy.

## Introduction

Atrial fibrillation is a common supraventricular arrhythmia with increased risk of stroke and heart failure [1–3]. Atrial fibrillation is most often diagnosed by an irregular rhythm and absent P waves (or rapid irregular fibrillatory waves) on the 12-lead ECG [1]. Regarding anticoagulant therapy, atrial flutter is managed the same way as atrial fibrillation [1]. An incorrect diagnosis of either atrial fibrillation or atrial flutter exposes the patient to potentially harmful treatment, for example, anti-coagulant therapy, as well as unnecessary further tests [4]. Computerized ECG interpretation is implemented in most modern ECG machines and has been shown to decrease physician ECG interpretation time [5,6]. However, misclassification of arrhythmias is not uncommon using computerized ECG interpretation [4,6–11]. For example, sinus arrhythmia, 2nd degree AV block Mobitz type I, extra-systolic beats, atrial tachycardia or artifacts may result in false diagnoses of atrial fibrillation or atrial flutter [4,8]. It is important that the physician corrects an erroneous computer-based diagnosis of atrial fibrillation or atrial flutter to avoid unnecessary anticoagulant treatment.

Patients with atrial fibrillation or atrial flutter are commonly encountered in primary care but data regarding the incidence of misdiagnoses in primary care settings are lacking. In Sweden, almost all primary care facilities use computer-based ECG interpretation, and it is common practice to record ECGs in patients consulting for various reasons (e.g. hypertension, dizziness, fainting, chest pain or dyspnea).

We aimed to describe the incidence of incorrect, computerized ECG interpretations of atrial fibrillation and atrial flutter in a Swedish primary care population, the rate of correction of computer misinterpretations and the consequences of misdiagnosis.

## Methods

Region Kronoberg in south Sweden serves the health-care needs of approximately 200,000 inhabitants through 31 primary health care centers that all use the same ECG recording system and central digital storage (EC store, Cardiolex AB, Täby, Sweden). All ECGs recorded between January 2016 and June 2016 at all primary health care centers in Region Kronoberg, with a diagnosis of either atrial fibrillation or atrial flutter, suggested by the built-in ECG interpretation program (University of Glasgow ECG analysis program, version 28.5.1), were included in this study. ECGs were retrieved from the central digital ECG database and included if either the term ‘atrial fibrillation’ or ‘atrial flutter’ was mentioned in the computer interpretation report. If several ECGs were identified for the same patient, only the first ECG was included. Patients younger than 18 years were excluded.

For the purpose of this study, atrial fibrillation was defined as an irregular supraventricular rhythm *and* absence of discernible P waves. Atrial flutter was defined as a supraventricular rhythm with regular flutter waves (F waves) with an atrial rate of 200–340/min and absence of an isoelectric baseline between discernible F waves. ECGs were re-assessed by one experienced ECG reader (TL, >10 years of experience of ECG interpretation including computer interpretation overreading) and one expert ECG reader (OP, >30 years of experience). ECGs with an incorrect diagnosis of atrial fibrillation or atrial flutter were assessed also by a third reader with vast experience in invasive electrophysiology studies, including ablation treatment for atrial fibrillation and atrial flutter (EL, >20 years of experience). A definitive rhythm diagnosis was determined by consensus. ECGs with an incorrect diagnosis were assessed for signal quality and for each ECG presence of either no, minor or major signal disturbances were noted.

Patient records for those patients who had an incorrect computer-based diagnosis of atrial fibrillation or atrial flutter were studied to find out whether (a) the incorrect computer diagnosis was corrected; (b) treatment with anti-coagulant therapy was initiated; (c) anti-arrhythmic therapy was initiated; (d) the patient was admitted to a hospital or (e) the patient was referred for follow-up tests. Data on b–e were not included if reasons other than atrial fibrillation or flutter were present for initiation of medical therapy (b, c), admittance to the hospital (d) or follow-up investigations were present (e). If the primary care physician interpreted an ECG with a computer diagnosis of atrial fibrillation as atrial flutter or vice versa, the diagnosis was considered to be *correct* in this study.

The study was approved by the Ethical Review Board in Linköping, Sweden (Dnr 2017/356-31).

### Statistical methods

Data are presented as mean (standard deviation) when appropriate. *χ*^2^ test was used to assess differences in computer misinterpretation of atrial fibrillation versus atrial flutter. A *p* value <.05 was considered statistically significant.

## Results

Between January 2016 and June 2016, 15,799 ECGs from 13,011 unique patients were recorded at the primary health care centers in Region Kronoberg. Among these, ECGs from 988 patients (582 men, 406 women), mean age 77.9 (10.6, range 19–95) years with atrial fibrillation or atrial flutter according to the computer interpretation were included. Among the diagnostic statements, the term ‘atrial fibrillation’ was present in 908 ECGs, and the term ‘atrial flutter’ in 80. In 846 out of 908 (93%), the computer interpretations of atrial fibrillation were correct, and 53 out of 80 (66%) computer interpretations of atrial flutter were correct, i.e. 7% of computer suggestions of atrial fibrillation were incorrect and 34% of computer suggestions of atrial flutter were incorrect (*p*<.001). The overall positive predictive value was 91%. In total, 89 patients (9%) did not have atrial fibrillation or atrial flutter according to the ECG re-assessment.

**Figure 1.**
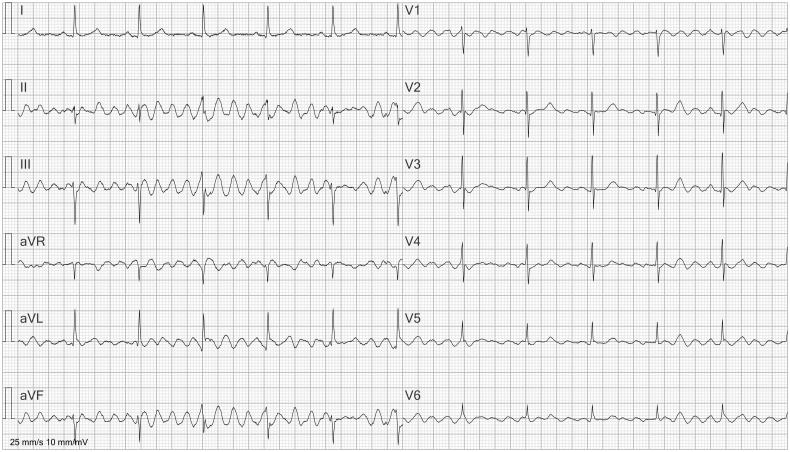
ECG interpreted as atrial flutter. Artifacts due to patient movement are present, interpreted as flutter waves by the computer algorithm. Absence of artifacts in lead I reveals that they are caused by movements of the left leg. This is because lead I measures the potential difference between the left arm and the right arm; the left leg is not involved.

The correct rhythm diagnoses are presented separately for atrial fibrillation and atrial flutter in Table 1. For both atrial fibrillation and atrial flutter, the most common cause of an irregular rhythm in the misinterpreted ECGs was either supraventricular or ventricular extra-systoles, accounting for 53% of the cases. Minor signal quality disturbances were present in 27 (29%) of the misclassified computer-interpreted ECGs, and major artifacts in 9 (10%) (Figure 1). Among those with sinus rhythm without premature beats or other cause of irregularity of the rhythm, at least minor disturbances were present in 50% (8/16 patients), compared to 38% of the remaining ECGs (28/73 patients) (*p*=.07).

**Table 1.** Correct rhythm of 89 primary care patients with an ECG computer based erroneous interpretation of atrial fibrillation or atrial fibrillation after a post hoc review of three experts in electrophysiology.

	Computer rhythm interpretation		
	Atrial fibrillation	Atrial flutter	
Correct rhythm	*N* (%)	*N* (%)	*p*
Sinus rhythm	2 (3)	12 (44)	<.001
Sinus arrhythmia	5 (8)	1 (4)	.6
SR with sinus arrest	2 (3)	0 (0)	n/a
Sinus rhythm with PAC	33 (53)	2 (7)	<.001
Sinus rhythm with PVC	6 (10)	1 (4)	.3
Sinus rhythm with PVC and PAC	1 (2)	1 (4)	.5
Sinus rhythm with non-sustained SVT	1 (2)	0 (0)	n/a
Sinus rhythm with 2nd AV block	6 (10)	0 (0)	n/a
SVT (sustained)	2 (3)	9 (33)	<.001
VT	1 (2)	0 (0)	n/a
Atrial pacing with PAC	3 (5)	1 (4)	.8

PAC: premature atrial complexes; PVC: premature ventricular complexes; SVT: supraventricular tachycardia (including ectopic atrial tachycardia); VT: ventricular tachycardia.

*χ*^2^-test was used to compare differences between the interpretation of atrial fibrillation and atrial.

Patient records could be retrieved for 85 (96%) of the 89 patients. An ECG interpretation made by the primary care physician could be found in the patient records in 77 cases. Among those with an incorrect rhythm diagnosis, the most common reason for the visit to the primary care physician was a scheduled routine visit (hypertension, diabetes mellitus, cardiac condition, etc.; 49%), followed by chest pain (14%) and dyspnea (13%). Palpitations were the cause of the visit in only 3%. Other causes were fatigue (3%), dizziness (2%), other (15%; fever, diarrhea, memory loss, weight loss, unknown cause, etc.).

**Figure 2.**
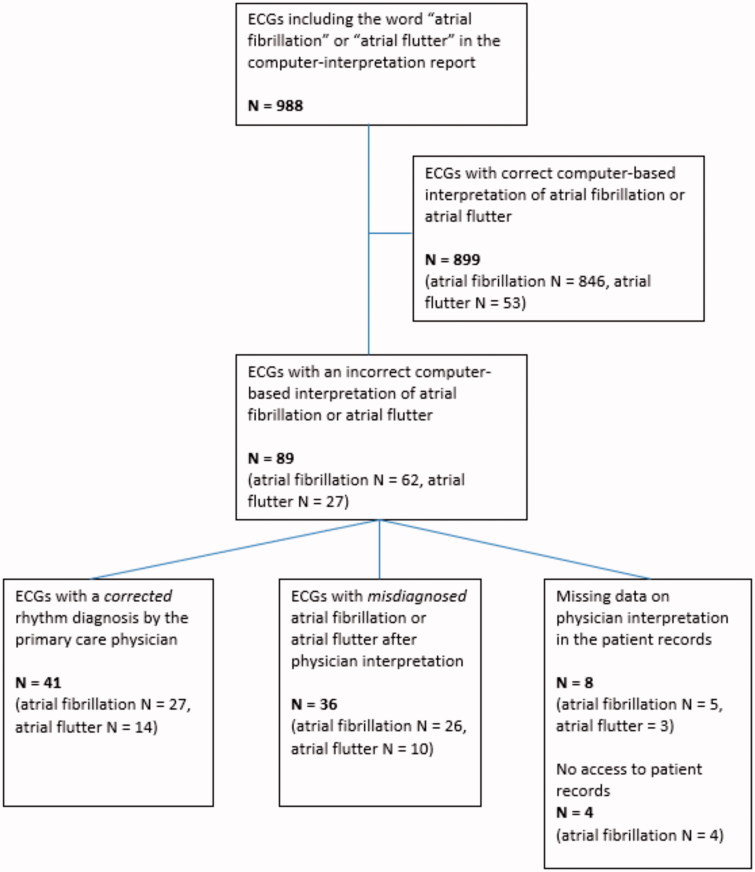
Flowchart describing the number of correct/incorrect computer-based interpretations, correction rate during primary care physician interpretation and missing data.

Among the 77 cases, where the interpretation made by the primary care physician could be found in the patient records, the erroneous computer interpretation was accepted by the interpreting physician in 36 (47%) of the cases and corrected in 41 cases (53%) (Figure 2). Anti-coagulant drug treatment was incorrectly started in 12 patients with misdiagnosed/over-called atrial fibrillation or atrial flutter, one patient received a beta-blocking agent and one patient received digoxin. Among those patients who were erroneously started on anti-coagulant therapy, no major bleeding events were found in the patient records and five of these patients received a correct ECG diagnosis during either follow-up or by the regional anticoagulation service facility. One patient was referred for cardioversion, 10 patients were referred to the emergency department and five patients were referred to an out-patient internal medicine department. Due to the (incorrect) finding of atrial fibrillation or atrial flutter, five patients were referred for echocardiography, three patients for Holter monitoring, one patient for exercise ECG while five patients were re-scheduled for a new appointment at the primary health care center. In nine cases, a cardiologist was consulted for ECG interpretation, resulting in a corrected diagnosis in all cases.

## Discussion

In this primary care study of computer-based ECG interpretations in a Swedish region false diagnosis of atrial fibrillation or atrial flutter by the built-in interpretation software occurred in 89 (9%) of 988 patients, i.e. the positive predictive value was 91%. In 36 cases, the erroneous interpretations were not corrected by the primary care physician. This eventually resulted in inappropriate anti-coagulant treatment in 12 patients. Atrial flutter was more often incorrectly diagnosed by the computer algorithm than atrial fibrillation.

A major limitation to this study is the retrospective design and the reliance on patient records to determine both the rate of correction of computer misinterpretations and ECG-based treatment decisions. However, information on ECG interpretation was missing in only eight cases. Another limitation is the small number of patients who received a misdiagnosis of either atrial fibrillation or atrial flutter, decreasing both the possibility to generalize the findings and to draw conclusions on major adverse events due to misdiagnosis. Furthermore, only one interpretation program was used, and the results may not be the same with other software. The strength of this study is that it comprises all ECGs recorded within the same geographical region and within the same primary care setting.

Although the issue of false-positive diagnoses of atrial fibrillation by computer software has been addressed previously [4,8,10,12], this is the first study that describes the incidence of misdiagnosis *and* correction rate in a primary health care setting. In a hospital-based setting, Bogun et al. [4] reported inaccurate computer interpretations (GE Marquette 12S or MACR program) of atrial fibrillation or atrial flutter in 19% of the cases. Of 382 ECGs in that study, 24% were not corrected by the physician who ordered the ECG, and clinical management was changed because of misdiagnosis in 42% of the patients. In a more recent hospital-based study, Hwan Bae et al. [8] reported over-interpretation of atrial fibrillation by the computer software (Philips 12-lead system) in 9% of the ECGs and a correction rate of 85%. In >4000 ECGs recorded at a university hospital, Poon et al. reported a PPV of 85% in computer-based interpretation of atrial fibrillation, and 83% in atrial flutter [10].

**Figure 3.**
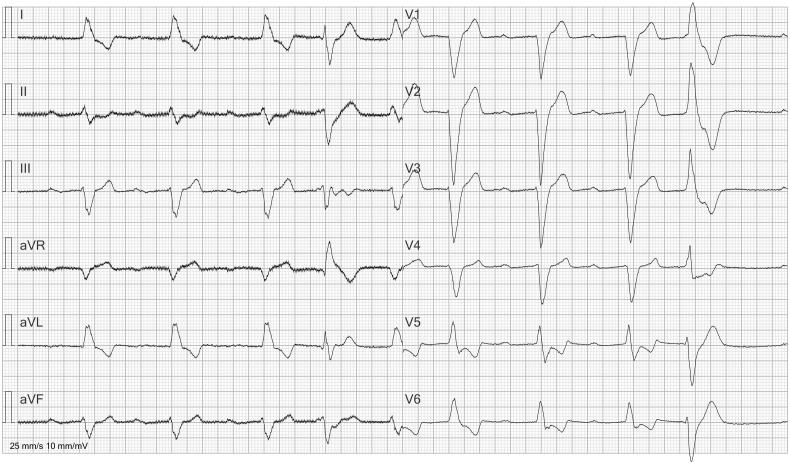
ECG with computer interpretation of atrial fibrillation. ECG shows sinus bradycardia and 1st degree AV block with a very long PR interval. The rhythm is irregular due to a premature ventricular contraction (4th beat).

**Figure 4.**
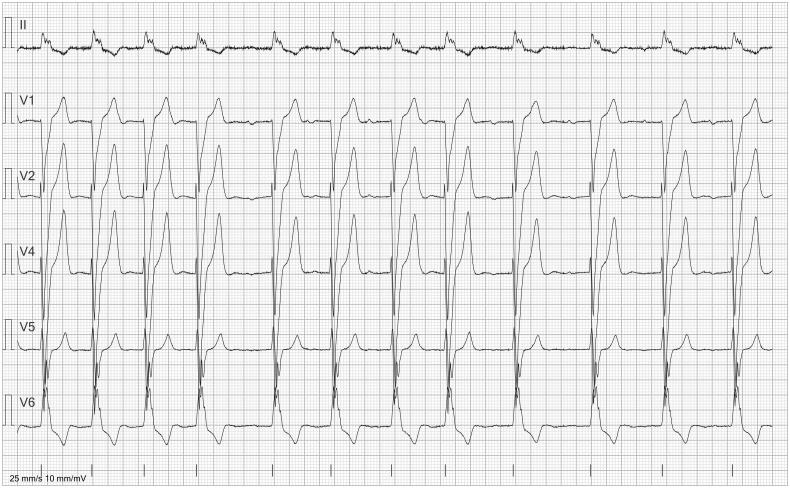
ECG in a 6-lead presentation (lead II, V1, V2, V4–V6). The ECG was incorrectly labeled as ‘atrial fibrillation’ by the automated report. The rhythm is irregular, but small P waves are visible (especially in V1) before most QRS complexes. The irregular rhythm is caused by a short run of premature supraventricular complexes and a premature atrial contraction.

Compared to our results, the rate of misinterpretation by the interpretation software was higher in the studies by Bogun et al. and Poon et al., but similar in the study by Hwan Bae et al. Correction rate was higher in the previous studies (76% (Bogun et al.) and 85% (Hwan Bae et al.)) compared to our study (53%) [4]. In contrast to previous papers, ECG interpretations in our study were performed by primary care physicians. ECG interpretation skills among primary care residents have been shown to be suboptimal and not to improve over time [13]. Several of the false atrial-fibrillation calls by the interpretation software were not easily detected (Figures 3 and 4). Given the very broad knowledge required in primary care, profound ECG interpretation skills cannot be expected. In the few cases (10%), when the primary care physician asked a cardiologist for a second opinion, false diagnoses were avoided. As in previous papers [4,8], inappropriate management was uncommon, and serious complications were rare. This study was performed during a short time period but considering the number of ECGs recorded each day worldwide and the number of misinterpreted ECGs, the risk of complications due to misinterpreted atrial fibrillation cannot be disregarded. Also, when a patient is given a certain diagnosis, it tends to ‘stick’ on the patient over time, and later management may be affected by a previous, incorrect diagnosis of atrial fibrillation or flutter.

Physicians are highly influenced by the diagnosis suggested by the computer decision support [6,14]. In a randomized controlled trial, Tsai et al. studied the accuracy of ECG interpretations made by internal medicine residents, with and without computer decision support. Study participants were given two sets (A and B) of equally difficult ECGs to interpret. One group interpreted set A without computer interpretation and set B with computer interpretation, whereas another group interpreted set B without computer interpretation and set A with computer interpretation. Two cardiologists also interpreted the ECGs and decided whether the computer interpretation was correct or not. When the computer diagnosis was correct, physicians interpreted the ECG correctly in 68% of cases with correct computer interpretation available as compared to 53% when correct computer interpretation was not available [14]. However, when the computer diagnosis was incorrect the number of correct physician interpretations was lower (48%), compared to 57% when the computer interpretation was not available.

In a study using simulated cases, Hillson et al. described that computerized ECG interpretation increased accuracy among primary care physicians in making the correct clinical diagnosis and shortened ECG interpretation time by 25%. In two of the ECGs given to the study participants, the computerized rhythm interpretation was incorrect, but corrected by only 24 and 10% of the physicians, respectively [6].

Detection of atrial flutter is based on both detectable P waves (or F waves), atrial rate (taking into out account F waves hidden within the QRS or T waves) and ventricular rate [15]. To our knowledge, there is no publication describing in detail the computer interpretation algorithm for atrial fibrillation in standard 12-lead resting ECGs. It is however likely that computer interpretation of atrial fibrillation is based on both absence of discernible P waves, or the presence of multiple, irregular ‘P waves’, and irregular R-R intervals. Extrasystoles were present in approximately half of the cases, similar to the results of previous studies [4,8]. Artifacts are also a common cause of misinterpretation [4,8,12]. In our study, major artifacts accounted for 10% of computer misinterpretations.

Although the rate of major bleeding events with anti-coagulant drug treatment is fairly low (2.9–4.3 in 100 person-years [9]), this risk is only acceptable if there is an accompanying expected decreased risk of stroke due to drug treatment. In our study, few patients with a mis-interpreted ECG started anti-coagulant drug treatment and none of them suffered a major bleeding event while they were being inadequately treated.

ECG over-reading has been proposed to overcome some of the pitfalls in computerized ECG interpretation [16,17]. In Sweden, all patients who are started on anti-coagulant drug treatment are referred to an anticoagulation service facility. Beside the need for improved ECG education in primary care, a possible safety-net solution to the problem of incorrect computer diagnosis and the low correction rate in our primary care setting is to make the anticoagulation service facility responsible for ECG over-reading performed by physicians highly skilled in ECG interpretation.

Computerized decision-support systems are developing fast, not only in ECG interpretation, for example in pulmonary function testing [18], dermatology [19], nuclear medicine [20] and radiology [21]. When the future of computerized decision-support systems is being discussed, computerized ECG interpretation is referred to as a success story [22], with substantial improvements since the first clinical use more than 30 years ago [23]. Nonetheless, as is shown in this study, rhythm disorders still pose a significant challenge, and physician over-reading is recommended [16,17].

## Conclusions

False-positive diagnosis occurred in almost one tenth of computer-based interpretations of atrial fibrillation or atrial flutter. In almost half of the cases, the erroneous diagnosis was not corrected by the over-reading primary care physician leading to inappropriate anticoagulant treatment in 12 patients.
